# How critical is SME financial literacy and digital financial access for financial and economic development in the expanded BRICS block?

**DOI:** 10.3389/fdata.2024.1448571

**Published:** 2024-12-19

**Authors:** Manoj Kumar M., Nasser Almuraqab, Immanuel Azaad Moonesar, Udo Christian Braendle, Ananth Rao

**Affiliations:** ^1^Department of Information Science and Engineering, Nitte Meenakshi Institute of Technology, Bangalore, India; ^2^Dubai Business School, University of Dubai, Dubai, United Arab Emirates; ^3^Mohammed Bin Rashid School of Government, Dubai, United Arab Emirates; ^4^IMC University of Applied Sciences Krems, Krems an der Donau, Lower Austria, Austria

**Keywords:** PCA, Random Forest Tree, digitalization, GMM, SME financial awareness, SDG 8, 9, 10

## Abstract

**Introduction:**

The expanded BRICS block presents significant opportunities for SMEs (Small and Medium Enterprises), but challenges related to financial literacy and digital access hinder their potential. While global efforts emphasize financial literacy and digitization as key drivers of economic growth, especially in developing regions, their specific impact on SMEs in the BRICS block remains underexplored. This paper contributes to the literature by contextualizing how financial literacy and digital financial access influence SME sustainability and economic progress, particularly in light of ongoing efforts to bridge the digital divide.

**Methods:**

Using Principal Component Analysis to reduce dimensionality, the study uses advanced Random Forest Tree modeling, to evaluate current practices in SME finance, credit access, and digitization.

**Results:**

Results indicate that both financial literacy and digitalization play pivotal roles in driving sustainable economic development, with significant implications for policy interventions aimed at supporting SME growth in emerging economies.

**Discussion:**

This study addresses the crucial intersection of SME financial literacy and digital financial access, focusing on their role in fostering economic development within the expanded BRICS block-a group now comprising major emerging economies that collectively face substantial disparities in financial inclusion. The study results are relevant not only for understanding the BRICS context but also for shaping global strategies toward inclusive financial systems and SME resilience in the digital era.

## 1 Introduction

Financial literacy has been recognized at a global level as a core life skill in the 21st century, one that is essential for the empowerment of individuals and for supporting individual and societies' financial well-being. The experience of the COVID-19 pandemic, increased cost-of-living pressures stemming from pandemic-related disruptions and Russia's invasion of Ukraine, and the current context of high inflation and rising interest rates highlight the continued need to strengthen financial literacy to support the financial well-being of individuals and households. Furthermore, the spread of digital financial services, which accelerated during the COVID-19 pandemic, underscores the need to equip individuals with adequate knowledge and skills to use such products and services safely. Other recent developments in the financial landscape, including a growing interest in and use of crypto-assets, new and alternative forms of financial advice (e.g., finfluencers), and the increased incidence and complexity of financial frauds and frauds also highlight the need to strengthen financial literacy skills among adults to help them make sound financial decisions. As stated by the OECD Recommendation on Financial Literacy, collecting dependable and internationally comparable data to measure the financial literacy of adults is a key step in creating successful national strategies and programmes for financial literacy (OECD, 2020, 2022, 2023). Such data can provide evidence of the areas in which financial knowledge and skills need improvement and the groups of adults who need financial literacy the most. Repeated measures of financial literacy also help identify trends and indicate where improvements have been made and what more needs to be done.

Governments and central banks of the **B**razil, **R**ussia, **I**ndia, **C**hina, and **S**outh Africa (BRICS) countries have undertaken several initiatives to ensure access and affordability of financial services to all sections of society, especially SMEs, weaker sections, and low-income groups. About 30 percent of the unbanked adults (1.7 billion globally) are in the BRICS countries as per the World Bank's Global Findex Report 2017. There is a considerable gap across generations and between the employed and unemployed sections of the population as well, with the younger generation (ages 15–24) and the population out of the labor force having lower account ownership. Leveraging digital tools can accelerate the reach and pace of financial inclusion. While the BRICS are rapidly employing digital technology, there still remains immense potential in furthering financial access through digital financial inclusion.

In this study, SMEs are categorized based on criteria established by the International Finance Corporation (IFC), which include the number of employees, annual sales, and total assets. These criteria are crucial as they allow for a standardized comparison across different economies within the expanded BRICS block (Table 1).

**Table 1:** Classification of small and medium enterprise (SME).

**Firm size**	**Employees**	**Assets (million US$)**	**Annual sales (million US$)**	**Loan size proxies (US$)**
Small (S)	10– < 50	0.1– < 3	0.1– < 3	10,000– < 100,000
Medium (M)	50– < 300	3– < 15	3– < 15	100,000– < 1 Million^*^

^*^ < USD 2 million for some advanced countries.

Female-Led Business: In addition to the above criteria, IFC classifies the SME as women-led or women-owned enterprise:

(A) If ≥ 51% firm's capital is owned by woman (women); **OR**

(B) If ≥ 20% firm's capital is owned by woman (women) **AND;** has at least one woman serving as CEO, or COO, or President, or Vice President; **OR**

(C) If ≥ 20% firm's capital is owned by woman (women) AND has ≥ 30% of the board of directors (if one is present) is made up of women.

(Source: https://www.ifc.org/en/what-we-do/sector-expertise/financial-institutions/definitions-of-targeted-sectors#).

In Table 1, IFC categorizes a firm as **Small (S)**:

- If it has between 10 and < 50 employees and annual sales of 0.1 to < 3 Million US$. **OR**
- If it has between 10 and < 50 employees and assets of 0.1 to < 3 Million US$. **OR**
- If it has between 10 and < 50 employees and borrowed 10 to < 100,000 US$.

Similarly, IFC categorizes a firm as **Medium (M)**:

- If it has between 50 and < 300 employees and annual sales of 3 to < 15 Million US$. **OR**
- If it has between 50 and < 300 employees and assets 3 to < 15 Million US$. **OR**
- If it has between 50 and < 300 employees and borrowed 100,000 to 1 Million US$.

In addition to meeting the above criteria, IFC categorizes the firm as **Women-owned Small**:

- If women own > 51% firm's capital; **OR**
- If women own > 20% firm's capital AND has at least one woman serving as CEO or COO, or President, or Vice President. **OR**
- If women own > 20% firm's capital AND women constitute > 30% of board of directors (if the firm has a BOD).

By dispersing wealth, creating jobs, developing technology, reducing poverty, empowering women, and encouraging entrepreneurship, the SME sector contributes to the economic prosperity of nations (Kwaku, 2018; Hossain et al., 2020). The working environment of small firms is complex, even if they provide jobs, and many owner-managers have a lot of difficulties when deciding how much money to spend on running their companies (Hossain et al., 2020). While it is necessary to manage business operations, SME financial literacy presents a problem (Hossain et al., 2020; Widiyati et al., 2018; Agyapong and Attram, 2019). As a result, entrepreneurs have made a major effort to learn financial literacy as they understand how important it is to the prosperity and reputation of their companies. Research has shown that financial literacy favors organizations' performance in terms of savings, revenue growth, and ease of access to capital for operating costs (Ali et al., 2018). As a result, many think that small firms' capacity to manage money is essential to their success. As long as rationality serves as the foundation for wise decision-making, financial management is probably going to be more effective if there is more SME financial literacy (Widiyati et al., 2018).

Digital financial access opens opportunities for SMEs, from enhanced access to credit and investment to streamlined operations and access to new markets. The adoption of digital financial services can significantly lower transaction costs, increase the efficiency of financial operations, and expand the reach of businesses beyond traditional geographic limitations. In the BRICS nations, where the penetration of traditional banking services can be limited, especially in rural and underdeveloped areas, digital financial platforms serve as a critical lifeline, providing SMEs with the tools necessary for growth and expansion (Khera et al., 2021).

The resource-based view holds that tangible and intangible resources are necessary for an organization to succeed and maintain a competitive edge (Das and Teng, 2000). Few people have focused on the impact of knowledge-based resources on the success of small businesses (Zhao, 2019). The knowledge base view states that one of the intangible resources that impact the viability of small firms is financial literacy (Jappelli and Padula, 2013). According to Zhao (2019), knowledge-based resources are thought to have a more significant influence on growth than material resources when pursuing an entrepreneurial approach and elucidating how businesses can develop and preserve advantages and capabilities that provide them a competitive edge. Yet, pooling these assets maintains a long-term competitive edge (Putra et al., 2021).

## 2 Research problem

The significance of financial literacy in ascertaining the sustainability of small enterprises is extensively acknowledged on a global scale (Eniola and Entebang, 2016). Its impact on obtaining financial services has been acknowledged (Okello et al., 2023; Ye and Kulathunga, 2019). Recent developments in small business finance highlight how important financial literacy is to entrepreneurs as a means of survival. The fact that inadequate financial knowledge can lead to poor financial decisions illustrates the significance of financial literacy (Eniola and Entebang, 2017; Refera et al., 2016).

While some argue that limited financial literacy among owner-managers may present challenges for small businesses seeking formal credit, potentially impeding their capacity to produce precise and superior financial reports (Buchdadi et al., 2020), most of empirical research has been devoted to assessing credit availability and managerial skills. In contrast, there has been comparatively little research on the importance of improving financial literacy, even though it is recognized as a critical element in promoting the growth of business (Azmi et al., 2020; Agyapong and Attram, 2019).

The BRICS countries—a group of quickly emerging economies—are facing the urgent problem of limited financial literacy among SMEs while also seeing tremendous technical advancement for SMEs. As a significant amount of the world's population resides in these countries, where technology has an impact on SME sustainable growth, it is critical to investigate the connections between SME financial literacy access to finance, SME digitization for sustainable financial and economic development of SMEs and in turn the respective BRICS countries. With the addition of six nations—Argentina, Egypt, Ethiopia, Iran, Saudi Arabia, and the United Arab Emirates as full members of the BRICS Summit in October 2023, the BRICS alliance currently includes 11 nations with a combined GDP of US$30.76 trillion, or 30% of the global economy, and 40% of the global population. The summit is delegates emphasized the BRICS block's cooperation to advance the Sustainable Development Goals (SDGs), decent work, and economic growth (SDG 8), industry, innovation, and infrastructure (SDG 9), Sustainable cities and communities (SDG 11) foster global recovery through Partnerships (SDG 17). They realized that the biggest global problem and a prerequisite for sustainable development is eradicating poverty in all its manifestations, including extreme poverty. While it is theoretically possible to reduce international differences through digitalization and financial inclusion by facilitating economic development and catching up, there is a chance that unintended consequences could arise, such as the escalation of already-existing disparities or the evolvement of new forms of income inequality during uncertain pandemics. Identifying determinants of income inequality is crucial to overcoming hindrances to future growth capacity (Iammarino et al., 2019).

**Figure 1:**
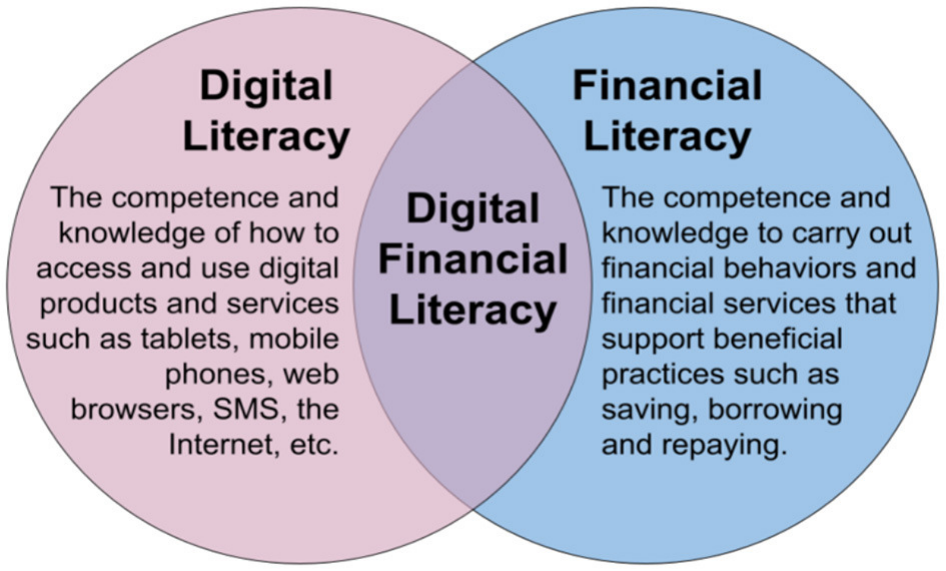
Interdependency of digital, financial, and digital financial literacy (https://www.futurelearn.com/).

Digital financial inclusion refers to the use of digital financial services to further the goal of financial inclusion. It aims to leverage digital means to reach out to the financially unserved and underserved populations with a basket of formal financial services and products suited to their needs in an affordable, safe, and transparent manner. At the same time, it promotes efficient and effective networking among participants. Any digital financial service is based primarily on three components, *viz*., digital transactional platforms, devices, and retail agents.^1^ Among others, the benefits of digital financial inclusion include: faster and wide-spread dissemination of formal financial services; equality of access to all; relatively lower costs of digital platforms as against physical “brick and mortar” models; availability of need-based, customized and better-priced products/services for diverse customers; convenience to the customers; lesser risks and costs associated with the handling of cash; and lastly, an opportunity for economic empowerment of women, youth and vulnerable sections of the society including SMEs. Figure 1 summarizes the interdependence of digital literacy, financial literacy and digital financial literacy skills needed to navigate financial services with the skills to use digital technologies.

A PwC Report estimates that the volume of cashless transactions will increase by 80 percent to 1.9 trillion by 2025, while digital payments per person will triple by 2030. Such transformations in the digital arena have led to significant re-engineering of the modes to enhance the financial inclusion of the unserved and the under-served population in the BRICS.

The BRICS Digital Financial Inclusion Report (2021) states that BRICS economies are facing the urgent problem of limited financial literacy among SMEs while also seeing tremendous technical advancement for SMEs. As a significant amount of the world's population resides in these countries, where technology has an impact on SME sustainable growth, it is critical to look into the connections between SME financial literacy access to finance, SME digitization, for sustainable financial and economic development of SMEs and in turn the respective BRICS countries.

In this background, this paper examines the important link between SMEs' education, access to finance and digitalization of SMEs for sustainable financial and economic development in the expanded BRICS alliance during pandemic events by answering specific research questions:

1. What is the role of financial literacy among SMEs in driving economic development within the expanded BRICS block?
2. How does access to formal credit financing impact SME growth and broader economic development in the expanded BRICS countries?
3. How does digital financial inclusion, through digitization and technological adoption, influence SME sustainability and macroeconomic outcomes in the BRICS block?

### 2.1 Research uniqueness and contributions

In the empirical literature, little research has investigated the connection between small business sustainability and SME financial literacy (Agyapong and Attram, 2019; Eniola and Entebang, 2016; Ye and Kulathunga, 2019). Nevertheless, these studies neglect to consider the role that formal credit availability plays in the relationship between the financial literacy of owner-managers and the small enterprises' ability to survive pandemics in the BRICS expansion. The current study advances our understanding of digitization, capital availability for small and medium-sized businesses (SMEs), and financial literacy.

It first focuses on a resource-based view and knowledge-based framework to underline the importance of SME access to formal finance and SME financial literacy in the small business sector during pandemic occurrences.

Second, unlike previous studies, the research contributes to the literature on small enterprises by utilizing Dynamic Panel Generalized Mixed Model (GMM) and Random Forest Tree (RFT).

Third, the study investigates small firm owner-managers SME financial literacy experimentally. This topic has not gotten much attention in the enlarged BRICS block.

Fourth, the article's empirical results may inspire policymakers to create initiatives to raise the financial literacy of entrepreneurs in expanded BRICS block's financial and economic development.

Fifth, it highlights the potential role of digital financial literacy in reducing income inequality by providing SMEs with the tools necessary to access and utilize financial services effectively, thereby fostering inclusive economic growth.

### 2.2 Uniqueness

To better understand how SME financial literacy, SME access to credit, and SME digitization drive SME well-being, as well as who experiences financial hardship, what causes it, what the short- and long-term consequences of it, and how SME attempts to mitigate it in the short term by borrowing money or depleting assets, a more significant amount of analytical work is needed at the global and regional block level. There is scant research on these issues in economic blocks such as the enlarged BRICS, which are connected to the established economies in the EU (represented by Austria). To assist policymakers in taking the necessary steps to meet the targets set under SDGs 8 (Decent Work and Economic Growth), 9 (Innovation and Infrastructure), and 17 (Partnerships for the Goals) in these economies by 2030, this study broadens the corpus of academic knowledge and practice. These characteristics add to the current research's distinctiveness.

Furthermore, by conducting this study, we will be adding a unique perspective on the enlarged BRICS countries, which are anticipated to have a major impact on global economic growth in the years to come; to the body of literature previously extensively written about SME's role in financial and economic development. Although numerous studies have examined how new technologies impact SMEs' adoption of digitalization, few academic publications focus solely on developing markets such as the expanded BRICS countries. Even fewer studies have examined the potential contribution of financial access to closing the financing gaps between these countries' SMEs and technical advancement as well as their financial awareness. Because of this, there is a significant lack of information about the influence in these areas and the possible roles that policy integration could play in either aggravating or decreasing this problem within the framework of the enlarged BRICS countries.

### 2.3 Innovation

This study uses Random Forest Tree (RFT) modeling, an artificial intelligence (AI) tool, to evaluate the data, and it shows that it is better than the usual econometric (Dynamic Panel GMM regression) approaches used in previous studies.

### 2.4 Significance

The following contributions to the significance of this research are included:

- The study first focuses on a resource-based view and knowledge-based framework to underline the importance of SME access to formal finance and SME financial literacy in the small business sector during pandemic occurrences.
- Second, unlike previous studies, the research contributes to the literature on small enterprises by utilizing Dynamic Panel GMM, and RFT, a machine learning technique for handling non-linear relationships using a trained set for prediction purposes.
- Third, the study investigates small firm owner-managers' SME financial literacy experimentally. This topic has not gotten much attention in the enlarged BRICS block.
- Finally, the article's empirical results may inspire policymakers to create initiatives to raise the financial literacy of SMEs in expanded BRICS block's financial and economic development.
- The results have Policy implications on

- SDG: 8: (Sustained Economic growth and Development through creating jobs by encouraging SMEs).
- SDG 9 (Digitization through Innovation and Infrastructure).
- SDG 10: (Reducing inequalities by Disbursing wealth and empowering women).

The upcoming contents of this paper are organized into six sections. Section 2 discusses our conceptual framework and reviews relevant literature. Section 3 details the Methodology and key data indicators and develops relevant hypotheses. Section 4 presents the results of statistical and RFT machine learning methods. Section 5 discusses the study findings. Section 6 concludes with implications for policy and practice with future research directions.

## 3 Theoretical underpinning, related research review, and hypothesis development

### 3.1 SME financial literacy

The literature on small businesses goes into considerable length about how loan availability and SME financial literacy affect a business's capacity to remain viable. Sustainability is a business and investment strategy that aims to meet and balance the needs of present and future stakeholders as efficiently as possible (Al-Abbadi and Rumman, 2023; Ismail, 2022; Latifah and Soewarno, 2023).

An enterprise's capabilities and resources are recognized as the main factors that determine its sustained competitive edge within the framework of resource-based theory (Madhani, 2009). Knowledge-based theory contend that there is a critical relationship between knowledge and enterprise performance, with data showing that knowledge—along with other resources unique to an enterprise, such as finance—is essential to success and long-term competitive benefit (Putra et al., 2021; Owusu et al., 2019). The creation and maintenance of competitive advantage in businesses is thus best explained by the combination of resource-based and knowledge-based views. According to recent studies, formal credit access and financial literacy for entrepreneurs are critical for small business sustainability (Ye and Kulathunga, 2019).

According to the knowledge-based perspective, SME financial literacy is a set of skills essential to small firms' ability to create value and experience long-term success. According to Das (2016), SME financial literacy is operationally defined as small business owner-managers' comprehension of fundamental economic concepts, ability to make wise financing decisions, and familiarity with legal and regulatory frameworks. Moreover, financial literacy is essential for sustaining a competitive edge and viability from a resource-based perspective because small enterprises in the modern economy need sufficient financing to run effectively (Hussain et al., 2018).

### 3.2 Digital financial literacy

Digital financial literacy does not stand in isolation but is a part of a broader ecosystem that includes digital infrastructure, regulatory frameworks, and the availability of digital financial services. The integration of these elements can catalyze the transformation of SMEs from traditional operations to more innovative and scalable business models. As such, SMEs' engagement with digital financial services is expected to enhance their financial inclusivity, thereby reducing the traditional barriers to credit access and financial services (Khera et al., 2021).

Within the small business literature, there is documented proof of how owner-managers' knowledge of finance influences the companies' profitability (Eniola and Entebang, 2016; Kimunduu et al., 2016; Kizza, 2019). The influence of financial literacy on loan availability for entrepreneurs is related to this effect (Dewi and Yurniwati, 2018); additionally, the viability of small businesses owned and controlled by entrepreneurs is impacted by formal credit availability (Kalaieesan, 2021; Patrick et al., 2021). Nevertheless, it is notable that limited information is available about the direct relationship between entrepreneurial literacy and business sustainability (Ye and Kulathunga, 2019).

According to the resource-based view, financial resources are necessary for small firms to grow and succeed (Kalaieesan, 2021). Funding access is a critical component of small businesses' long-term success. Because it enables them to experiment with new ideas, launch creative projects, and become more prepared to take advantage of opportunities (Dewi and Yurniwati, 2018). According to Nzibonera and Waggumbulizi (2020), financial access promotes profitability and growth while ensuring stability and sustainability. Small companies that take out external debt are better positioned for growth, and these companies' ability to obtain funding is crucial to their success (Patrick et al., 2021).

Entrepreneurial knowledge of finance is a skill set that improves the accessibility of financial services for overseeing enterprises in the knowledge-based economy (Dewi and Yurniwati, 2018). It is thought that greater financial literacy among entrepreneurs will help them make more educated decisions about financial services and increase the possibility that they will be able to acquire financing. On the other hand, small enterprises with low financial literacy could have trouble getting funding outside and generating reliable financial statements (Buchdadi et al., 2020). To increase finance accessibility and lower perceived risks connected to their companies, advisors advise proprietor-managers of small businesses to foster a financial literacy culture (Owusu et al., 2021). Higher financial literacy has been shown to promote financial inclusion and raise consumer demand for financial products (Khan et al., 2022). Financial literacy is necessary for making financial choices and achieving financial security (Pangestu and Karnadi, 2020). Financially knowledgeable business owners can attract more investors and readily take out external financing (Sulistianingsih and Santi, 2023).

### 3.3 Economic development in the expanded BRICS block

The economic development of emerging markets, particularly within the BRICS block has gained significant attention in recent years. SMEs play a pivotal role in these economies, acting as engines of growth, innovation, and employment. Financial literacy and digital financial access are critical factors influencing the success and sustainability of SMEs. This research investigates how these factors impact financial and economic development within the expanded BRICS block, employing advanced statistical and machine learning methods to uncover insights and draw meaningful conclusions.

SMEs are fundamental to the economic fabric of the BRICS nations, contributing substantially to GDP and employment. However, these enterprises often face significant challenges, including limited access to finance, inadequate financial literacy, and the need for digital transformation. Financial literacy equips SME owners and managers with the knowledge to make informed financial decisions, manage risks, and access various financial services. Digital financial access, on the other hand, leverages technology to provide SMEs with more efficient, scalable, and accessible financial solutions.

There are two schools of thought on how fiscal policy affects overall economic security. One theory holds that progress in the financial sector dampens volatility by lowering frictions and informational asymmetries; this, in turn, diminishes the impact of the financial accelerator on the amplification of cycles by making lending terms less sensitive to fluctuations in borrowers' net worth (Bernanke et al., 1999). One argument favoring financial growth is that it encourages risk-sharing, which in turn lessens financial limitations, makes consumers and businesses more resilient to shocks, and makes it easier to smooth out consumption. On the other hand, some argue that financial markets make crises more likely by encouraging risk-taking and leverage, especially in an unregulated and unsupervised financial sector. The knowledge-based perspective holds that an entrepreneur's ability to understand financial matters is essential to a small firm's launch, growth, and success. SMEs are better managed by entrepreneurs who have a basic understanding of economic fundamentals and financial literacy. Research indicates that these entrepreneurs can enhance the long-term sustainability of their businesses (Dewi and Yurniwati, 2018). The financial literacy of owner-managers is recognized globally as having a major impact on small enterprises' financial and economic stability and expansion (Prakash and Singla, 2022). Owner-managers can evaluate their demands, make wise financial choices, and enhance long-term performance with its help (Agyapong and Attram, 2019; Kizza, 2019; Usama and Yusoff, 2019). Furthermore, it is recognized that owner-managers' financial literacy improves business sustainability by encouraging ability development, financial literacy networking, leadership development, and mastery of fundamental business management skills (Eniola and Entebang, 2017). Additionally, research indicates that practices and knowledge related to financial literacy improve the sustainability of small-scale business activities (Grana-Alvarez et al., 2022; Babajide et al., 2021).

We can use conventional financing decisions predicated on the trade-off and pecking order theories to determine SME loan access. The trade-off between agency costs and the tax advantage by leveraging debt is termed as capital structure. The company's worth will only rise a little due to using debt. According to research by Jensen and Meckling (1976) and Myers and Majluf (1984), there is a negative correlation between capital structure and business value, suggesting that a rise in debt levels indicates a company's poor financial performance. The Trade-off theory requires businesses to employ loan capital as efficiently as possible by taking agency and bankruptcy expenses into account. Pecking order theory, as proposed by Luigi and Sorin (2009), reduces the requirement for outside funding by guiding the choice of corporate financing based on financing level. If retained earnings or other internal funding sources prove insufficient, external financing sources (debt or equity) will be used to fund the business. If required, they also select a funding source to reduce the added costs associated with asymmetric information.

In this sense, the only financial options available to SMEs are banking and non-banking financing. If SMEs follow the pecking order theory concerning capital structure theory, internal funding will be used to meet financing needs before turning to external financing if necessary (Delic et al., 2016). SMEs mostly depend on equity finance and bank loans to establish their businesses or fund their expansion. Obtaining finances from friends and family (FFF) or factoring is one of the less popular methods of obtaining capital. In developed financial markets, formal loans to small enterprises are difficult at this stage of their life cycle, but funds from friends and family are among the easiest sources of financing for startups. Supplier loans are another significant source of funding for businesses looking to expand, but they do not provide investors with enough legal protection. It is more likely that if a startup has physical assets that are used as security and have a legal form in the merger, it will include some external debt in its capital structure (Fourati and Affes, 2013; Karyadi and Rizki, 2018). Entrepreneurs who engage in more human capital-intensive businesses are more likely to be internally financed and have lower levels of external debt. Internal equity contributions are a more common source of funding for home-based endeavors. More educated businesspeople draw in more investors and have higher levels of external debt.

Alongside the biggest and most powerful banks, other non-bank financial institutions now hold important positions in the financial industry. Investment banks, insurance providers, mutual funds, pension funds, venture capital firms, and several others are among them. Similarly, the growth of financial markets has allowed people to diversify their assets and allowed companies to raise cash by selling stocks, bonds, and foreign exchange. Financial services are easier to supply when these FIs are in order. As a result, the effectiveness and availability of these financial services affect the level and rate of economic success. For SMEs, knowledge of finance is critical. According to Eniola and Entebang's (2017) research, financial performance is influenced by financial awareness, which suggests that managers and owners of businesses are aware of good financial services and will not pursue outside funding if it is hard to obtain. The present discourse is consistent with the wider themes expounded in the literature review and theoretical framework. According to Putra et al. (2021), the interplay among financial literacy, credit accessibility, and digital transformation is a crucial element in SMEs' sustainable expansion and competitiveness in the digital era.

With the advent of digital transformation, riding the tide of information technology, a significant overhaul is taking place in the fields of corporate strategies, company models, innovation policies, and marketing tactics (Verhoef et al., 2021). The digitization theory, backed by data, contends that changes in the global market and socioeconomic landscape are forcing SMEs to go digital. The current global issues exemplify how numerous business risks and uncertainties, such as pandemics, can disrupt global supply chains and accelerate the speed of digital transformation (Thanos et al., 2021). SMEs are forced to modify their business models and strategies due to various factors, including probable recession, related uncertainty, and inflation (Kraus et al., 2022; Marinko et al., 2021; Xie et al., 2022). Additionally, the end of the low-interest rate period is evolving, which benefits obtaining financing. These days, business models and digital revolutions are being greatly accelerated by the energy crisis and the war in Ukraine.

Previous studies have looked for clear trends in the adoption of Industry 4.0, with a particular emphasis on the front-end and basic technology layers. Businesses that are leading the way in implementing Industry 4.0 typically use a wide range of front-end technologies instead of focusing on just one or two of them (Matarazzo et al., 2021). The traditional business models of SMEs and the process of creating value for consumers are changing because of digital revolution (Müller et al., 2020).

SMEs in Europe are not exempt from the consequences of the digital revolution. Customer interactions are a common focus of studies on digital transformation in SMEs. These SMEs use digital technology to grow their customer base and develop and enhance overall business success through digital products and services (Khin and Ho, 2018). By using high-speed networks to communicate with suppliers and customers, SMEs and entrepreneurs can obtain real-time information and quickly adjust to rapidly changing supply chains and markets (Kergroach, 2021). SMEs' overall performance is enhanced, and their consumer base is increased with technology to create new digital goods and services (Khin and Ho, 2018). Furthermore, companies frequently deploy remote specialists and digital assistants to manage customer needs (Salvatore et al., 2016). In addition to enhancing existing operational work, digital transformation lowers input costs and increases production efficiency (Gal et al., 2019). SMEs that undergo a digital transformation see enhanced productivity, higher worker production, and better business outcomes (Hossain et al., 2020). Moreover, digital transformation facilitates novel financial management and payment approaches, enhancing SMEs' ability to secure financing and contributing to increased financial inclusion (Shofawati, 2019).

The rate and degree of digitalization of SMEs are closely related to Europe's competitiveness in international markets. The technology sector is growing at a rate that is five times faster than the overall European economy, causing SMEs to compete fiercely, scale their operations, and disrupt industries in unthinkable ways only a few years ago (Garzoni et al., 2020). Most studies on digital transformation that have been published concentrate on how these modifications impact value generation and structure, how digital technologies are used, how dynamic capabilities are, how consumers behave, and how strategic responses are used (Kraus et al., 2022).

**Figure 2:**
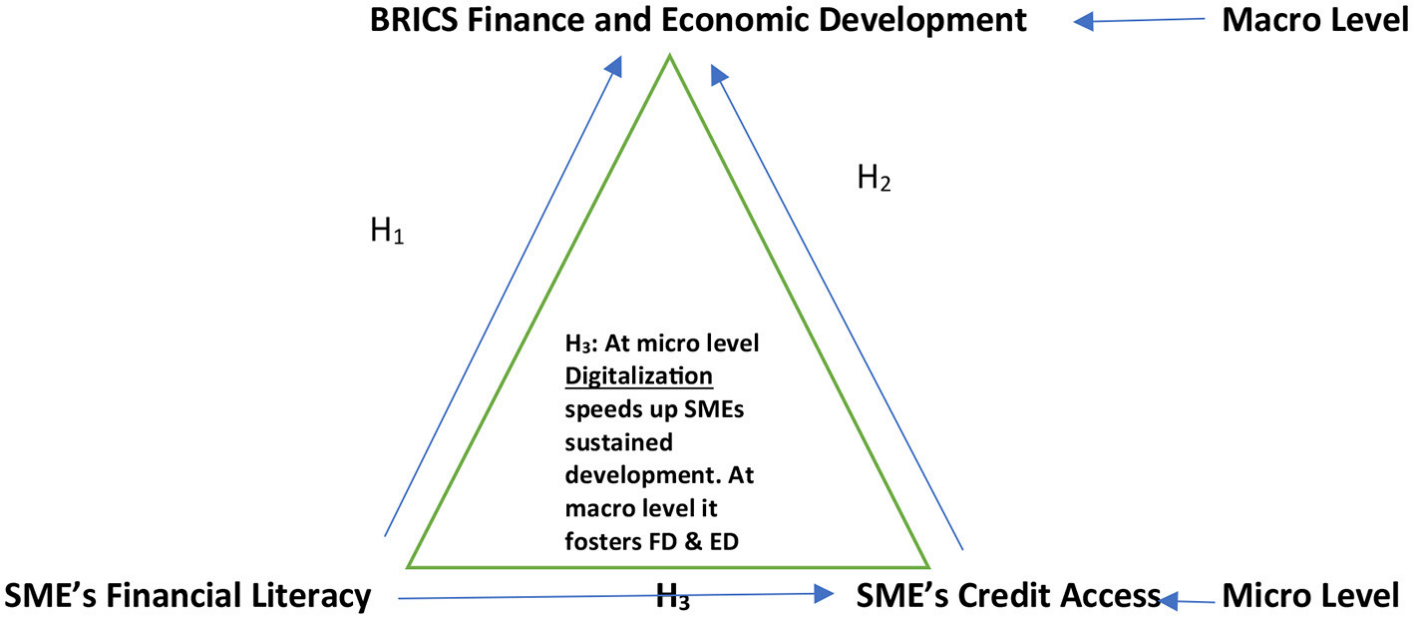
Financial literacy and loan availability of SMEs for the BRICS Financial and Economic Development Conceptual Framework using FINDEX Database. Global Findex Database 2017 of IMF is a comprehensive database constructed from survey data covering almost 150,000 people in 144 economies—representing more than 97 percent of the world's population. Gallup, Inc carries out the survey. The target population is the entire civilian, noninstitutionalized population age 15 and above. We chose to retain the Findex database due to its comprehensive nature, which aligns with our study's goal of a broad analysis of digital financial access. Findex Database Measures Financial Inclusion and the Fintech Revolution (Washington, DC: World Bank, 2018). This survey is longitudinal and is available over 2013, 2017, 2021 and hence help evaluate the trend of indicators in our research problem. No other platforms provide such a comprehensive data. Most researchers use the Findex database as their preferred set for addressing research problems covering various facets. For more details of these research please see Annex-1.Given these advantages, authors prefer to retain the Findex database for comprehensive analysis of digital financial access.

The framework shown in Figure 2 addresses the research questions using the preceding theoretical foundations. In Figure 2, the research postulates that:

**H**_**1**_: Expanded BRICS' financial and economic development are positively impacted by SMEs' financial literacy.
**H**_**2**_: SME's access to credit financing positively impacts expanded BRICS' financial and economic development.
**H**_**3**_**:** Digitization drives SME's financial literacy and access to credit, fostering the financial and economic development of expanded BRICS countries.

The following conventional econometric and artificial intelligence specifications are used to empirically examine the conceptual framework (see Equation 1):


(1) $$\begin{array}{c} Y_{\text{ijt}}=\alpha_{\text{ijt}}+\mu_{\text{i}}Y_{\text{i}-\text{kjt}}+\beta_{\text{ijt}}\sum \sum X_{\text{i}-\text{kjt}}+\pi_{\text{ijt}}\sum \sum Z_{\text{i}-\text{kjt}}\\ \;+\varepsilon_{\text{it}} \end{array}$$

Where:

Y_ijt_ = output = Financial and Economic Development Indices in expanded BRICS and Austria (i=12 countries, j=1 denote expanded BRICS and 0 otherwise; t = 2004-2022).

Y_i − kjt_ = k-period lagged Financial and Economic Development Indices.

X_i − kjt_ = Set of k-period lagged Input indicators covering SMEs' Financial Awareness, SMEs' credit access, and Digitalization inputs for expanded BRICS countries t = 2004-2022.

Z_i − kjt_ = Set of k-period lagged macro factors for expanded BRICS countries and t = 2004–2022 that population size, GDP, and inflation represent. This covers financial crisis events in 2008–2009 and pandemics (like COVID-19) that occurred between 2004 and 2020. We think that these occurrences have a more erratic and herd-like effect on SMES' sustainability and well-being in the study area due to its disruption of normal economic activity thus affecting income income-earning capacity of the vulnerable populace.

α, μ, β and π are coefficients respectively denoting intercept term to reflect fixed effect effects, Parameter coefficients for k-period lagged output, k-period lagged input and k-period lagged control factors.

We use the Dynamic Panel GMM Model with k-period lagged output, input, and control factors to allow for gradual adjustment over k-years, and Machine Learning (ML) Methods of estimation for the model parameters. These are discussed in Section 3.

## 4 Methodology, data, and indicators

We have chosen the following indicators developed by the IMF to gauge different elements of the Financial (F) index: Depth (D) of Financial Institutions (FI); Access of Financial Institutions (FIA); Development of Financial Markets (FMD); Access of Financial Markets (FMA); and Financial Markets Efficiency (FME).^2^ Six sub-indices produced by aggregating indicators are located at the pyramid's base. The weights obtained by principal component analysis (PCA) represent the contribution of each underlying series to the variance in the sub-index. These weights are then utilized to compute the aggregate, a weighted average of the underlying series (Sahay et al., 2015). Stronger institutions, which include better informational, creditor, and property rights protection as well as improved regulatory quality and rule of law, are positively connected with greater financial growth. The following definitions of FI depth, access, and efficiency are provided by Sahay et al. (2015).^3^

The accessibility of financial services to individuals and companies, the depth (scale and liquidity of markets), and the efficiency (capacity of institutions to offer financial services at reasonable costs and with steady income streams, as well as the volume of activity in capital markets) of the financial system of financial development (FD) are combined.

The variables used to gauge the depth of financial institutions (FI) are GDP to private sector credit, GDP to pension fund assets; GDP to mutual fund assets; and GDP to insurance premiums (both life and non-life). Access indicators include the number of bank branches and ATMs per 100,000 adults. The following measures are used to measure efficiency: ROA, ROE, non-interest income relative to total income, net interest margin, lending-deposits spread, and total assets incurred minus overhead.

**Figure 3:**
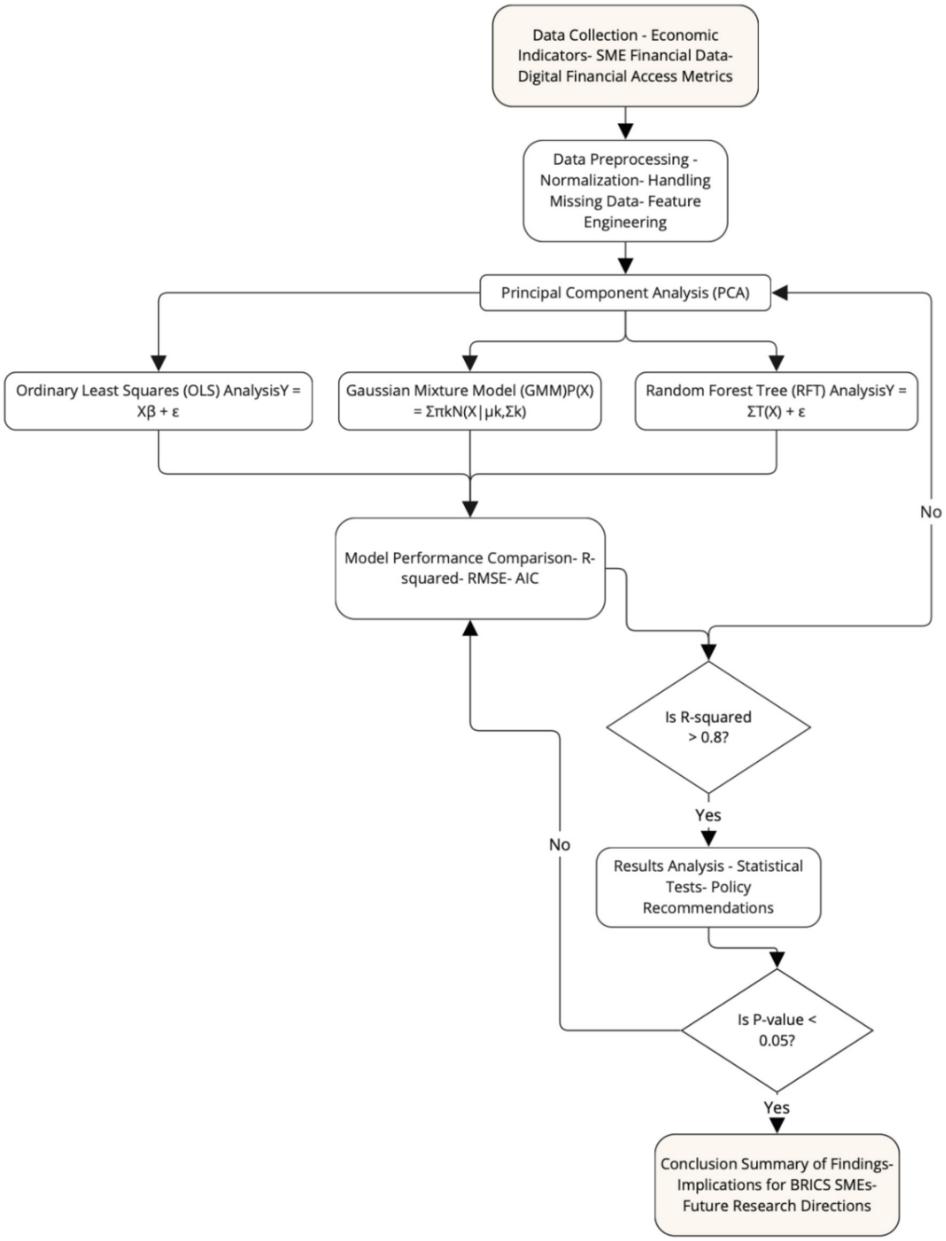
Research design and methodology.

Figure 3 illustrates the comprehensive research methodology employed to assess the impact of SME financial literacy and digital financial access on economic development within the expanded BRICS block. The process begins with Data Collection, where essential data is gathered from various sources, including economic indicators such as GDP and inflation rates, SME financial data like literacy scores and access to credit, and digital financial access metrics, including mobile money transactions and the availability of ATMs.

Following data collection, the Data Preprocessing phase involves preparing the data for analysis. This includes steps such as normalization, handling missing data, and feature engineering to ensure that the data is suitable for further statistical analysis. Principal Component Analysis (PCA) is then applied to reduce the dimensionality of the data, allowing the most significant features to be identified.

The next steps involve applying different modeling techniques:

- Ordinary Least Squares (OLS) analysis, represented by the equation Y=X β +ϵ (Y = X β + ϵ), is used to explore linear relationships between variables.
- The Gaussian Mixture Model (GMM), expressed as P(X) = ∑πkN (X|μk, Σk) P(X) = ∑π_*kN*(*X*|μ_*k*, Σ_*k*)*P*(*X*) = ∑πkN(X|μk,Σk), handles the heterogeneity in the data, allowing for the identification of latent variables.
- Random Forest Tree (RFT) Analysis, represented by Y = ∑T(X)+ϵY = ∑ T(X) + ϵ, captures complex non-linear relationships and the importance of different variables.

Once the models are applied, the **Model Performance Comparison** stage evaluates the models based on metrics such as R-squared, RMSE, and AIC to determine the best-performing model. A decision point then assesses whether the R-squared value exceeds 0.8, guiding the process toward results analysis if the criterion is met. If not, the data may be reprocessed using PCA.

In the **Results Analysis** phase, the selected model's outputs are examined through statistical tests, leading to policy recommendations. Another decision point checks if the p-value is less than 0.05, which, if true, advances to the **Conclusion** stage. Here, the findings are summarized, implications for SMEs in the BRICS block are discussed, and directions for future research are outlined. This structured methodology ensures a rigorous analysis of the factors influencing economic development in the BRICS context, with a focus on SME financial literacy and digital access.

### 4.1 Principal component analysis (PCA)

PCA is an unsupervised learning algorithm technique. Regression analysis, which finds the line of greatest fit, is often referred to as generic factor analysis. PCA is the machine learning methodology used for the FD index to avoid making assumptions about the significance of the indicators used to measure FD. Collinear indicators are combined in principle component analysis (PCA) to produce a composite indicator that includes the data shared by all the indicators. Our goal is to capture as much variance as possible in the set of indicators with the fewest possible parameters. As such, the composite index is now based on the data's statistical dimensions rather than the data set's dimensionality. The normalized series is averaged, and weights that are squared factor loadings (the sum of which must equal one), obtained from the PCA of the original series are used to construct the sub-indices. Factor loadings are coefficients that establish a connection between the factors or key components and the observed variables.

The square of a factor loading represents the factor's capacity to explain the overall unit variance of the indicator. The series that aids in identifying the direction of the common variation in the data is given greater weight. Weighting removes superfluous data points from numerous connected indications; it makes no evaluation of the linked indicator's theoretical relevance. PCA's primary objective is to decrease a dataset's dimensionality without affecting the target variables beforehand while maintaining the most significant patterns or links between the variables. Using PCA, a data collection's dimensionality can be decreased by identifying a new set of variables that are less than the original set while still containing most of the sample's information and being helpful for data regression and classification. The following are the characteristics of PCA:

- Arranged in decreasing order of significance, the principal components (PC) are linear combinations of the original variables in the dataset. All the PCs' combined variance captures add up to the same overall variance as the original dataset.
- According to **Table 3** through 5 in Section 4, the first PC records the greatest variation in the data, whereas the second PC records the biggest variance orthogonal to the first PC, and so on.
- PCA operates under the assumption that information is encoded in feature variance; that is, that a feature's larger variation, the more information it contains.

**The rationale for using PCA** (Principal Component Analysis) **Methodology** in this model: PCA reduces the number of variables or features in a data set while still preserving the most important information like major trends or patterns. This reduction can decrease the time needed to train a machine learning model and helps avoid overfitting in a model. These principal component values are “Eigen Values” derived from the Covariance matrix of a standardized data set with a mean of 0 and Standard deviation of 1. PCA can help us improve performance at a meager cost of model accuracy. Other benefits of PCA include reduction of noise in the data, feature selection (to a certain extent), and the ability to produce independent, uncorrelated features of the data.

Similarly modeling the impact of digitization and availability of credit on SME sustainable growth requires sophisticated statistical and ML techniques to capture the complexity of economic and social interactions. Three notable methodologies for conducting such an analysis are Ordinary Least Squares (OLS), Gaussian Mixture Model (GMM), and Random Forest, each offering unique advantages and insights. In our analysis framework, the model to be estimated is represented as in Equation 1.

**OLS** is a foundational econometric technique that estimates the relationship between dependent and independent variables by minimizing the sum of squared differences between the observed and predicted values. In the context of digitally enabled microfinance, OLS can provide initial insights into the linear associations between the availability and use of digital financial services and various outcomes, adjusting for other factors.

### 4.2 Gaussian Mixture Model (GMM)

Gaussian Mixture Model (GMM) is a probabilistic model for representing the presence of subpopulations within an overall population without requiring knowing which subpopulation a data point belongs to. GMM offers a robust approach to understanding the complex dynamics of digitally enabled microfinance and its impact on income equality, especially within the heterogeneous and multifaceted contexts of the BRICS block and Austria. The inherent heterogeneity of microfinance impact data, characterized by its origin from diverse sources and multiple underlying patterns or distributions, is well accommodated by GMMs. Additionally, GMMs are invaluable for uncovering latent variables that, while not directly observable, significantly influence observed outcomes. Examples include the level of digital literacy and access to digital infrastructure, as well as factors critical to the effectiveness of microfinance in fostering income equality. The flexibility of GMMs extends to the accommodation of diverse distribution shapes and sizes, recognizing that the impact of digitally enabled microfinance does not conform to a uniform pattern across all countries. By allowing for the modeling of mixture components with distinct means and variances, GMMs provide a nuanced and adaptable framework for analyzing the varied effects of microfinance initiatives, thereby offering deeper insights into strategies for mitigating income equality among vulnerable populations in emerging economies. Further, GMM is particularly suited for dealing with endogeneity issues, which arise when independent variables are correlated with the error term. GMM uses instrumental variables to tackle this problem, making it a powerful tool for causal inference in the study of digitally enabled microfinance. It allows a more nuanced understanding of the dynamic relationships between digital financial inclusion and economic outcomes over time.

### 4.3 Motivation for using GMM (Gaussian mixture model) methodology

One hint that data might follow a mixture model is that the data looks multimodal, i.e. there is more than one “peak” in the distribution of data. As shown in the example below, trying to fit a multimodal distribution with an unimodal (one “peak”) model will generally give a poor fit.

“Speeds across cars just before reaching a specific traffic light have multi modes before reaching the destination”. This is because traffic lights are not under the control of car drivers. Some drivers get green signal just like that, and some get red light depending on “traffic congestion”. Hence, the data set distribution of this example is most likely to be well-modeled using a Gaussian mixture model (GMM).

Since many simple distributions are unimodal, an obvious way to model a multimodal distribution would be to assume that multiple unimodal distributions generate it. For several theoretical reasons, the most commonly used distribution in modeling real-world unimodal data is the Gaussian distribution. Thus, modeling multimodal data as a mixture of many unimodal Gaussian distributions makes intuitive sense. Furthermore, GMMs maintain many of the theoretical and computational benefits of Gaussian models, making them practical for efficiently modeling very large datasets.

### 4.4 Decision tree model

The utility of decision trees in analyzing the impact of digitally enabled microfinance in the BRICS block, and Austria spans across several pivotal aspects. Firstly, their simplicity and interpretability stand out, making these models highly accessible to stakeholders with diverse technical backgrounds. This feature is particularly beneficial in the multifaceted realm of digitally enabled microfinance, where understanding the interplay of various factors leading to income equality reduction is crucial. Secondly, decision trees adeptly manage qualitative and quantitative data, accommodating the diverse data types inherent in microfinance analysis—from the digital platforms and regulatory environments to loan repayment rates and income levels. Thirdly, a significant advantage of decision trees lies in their ability to pinpoint the most critical variables affecting an outcome. Within the context of digitally enabled microfinance, this means identifying key factors like digital service accessibility, regulatory frameworks, and financial literacy levels instrumental in mitigating income equality. Fourthly, decision trees are equipped to model non-linear relationships, capturing the complex non-linear interactions between variables without the prerequisite of linear assumptions. This capability is vital for reflecting the nuanced real-world effects of microfinance on income disparity. Fifthly, decision trees offer unparalleled flexibility in scenario analysis, enabling a thorough exploration of the potential impacts of varying microfinance implementation strategies. Stakeholders can adjust variables and thresholds within the model to forecast the consequences of different policy decisions, technological developments, or market dynamics. This adaptability is invaluable for policymakers and analysts aiming to optimize digitally enabled microfinance programs for maximal effectiveness in reducing income equality across the blocks. Through these features, decision trees emerge as a powerful analytical tool, offering deep insights into the pathways through which digitally enabled microfinance can achieve its socio-economic objectives.

Random Forest Tree (RFT) is a machine learning approach that builds multiple decision trees and merges their predictions to improve accuracy and control over-fitting. This method effectively handles non-linear relationships and interactions between variables without requiring explicit specification. In analyzing digitally enabled microfinance, Random Forest can uncover complex patterns and variable importance, providing insights beyond traditional econometric models.

### 4.5 Motivation for using RFT (Random Forest Tree model) methodology

Data scientists use Random forest on the job in many industries including banking, stock trading, medicine, and e-commerce. It is used to predict the things that help these industries run efficiently, such as customer activity, patient history, and safety. Random Forest is used in banking to detect customers who are more likely to repay their debt on time. It is also used to predict who will use a bank's services more frequently. They even use it to detect fraud. Stock traders use Random Forest to predict a stock's future behavior. Retail companies use it to recommend products and predict customer satisfaction as well. Scientists use Random Forest to study the spontaneous combustion patterns of coal to reduce safety risks in coal mines. In healthcare, Random Forest can be used to analyze a patient's medical history to identify diseases. Pharmaceutical scientists use Random Forest to identify a medication's correct combination of components or predict drug sensitivity. Sometimes, random forests are even used for computational biology and the study of genetics.

The novelty of an approach should neither be taken as an argument in favor nor against using a certain modeling approach. The essential question is: Can the tool help us answer the question we have set out to address? The following are justifications for using ML in science:

**Time efficiency**: Accurate ML models can often be built time-efficiently by researchers. Less pre-processing is needed compared to other approaches and large parts of the model selection and training process can be automated. Also, there is great support for ML packages in popular programming languages like Python (e.g., scikit-learn, PyTorch, or TensorFlow) or R (e.g., mlr3 or tidymodels).

**Computational efficiency**: ML can be computationally cheaper than traditional modeling in some situations. ML weather prediction can run on your laptop, unlike numerical methods that demand the world's biggest computing clusters (Lam et al., 2023).

**The basis for theory**: ML may support us with the knowledge needed to build classical theory-based models. They may show which features contain predictive information and how features interact.

**Effective for operationalized goals**: ML is ideal if our aim can be easily encoded in a single metric. If we are confident in our metric, ML will provide us with the means to optimize for it.

**Theoretical underpinnings**: Theory starts to catch up to practical success slowly. For many methods, we can provide learning guarantees and even for deep learning techniques we increasingly have formal intuitions about what makes them work (Belkin, 2021).

Table 2 summarizes the set of dependent (outputs) and independent indicators (inputs) with sub-themes. We employ two dependent variables: GDP per capita annual average growth rate (Y_1_) to proxy economic development, and a second alternative by Natural log of GDP per capita (2015 Constant).

**Table 2:** Summary of Output (Y_ijt_) and Inputs (X_i − kjt_ and Z_i − kjt_ ).

**Y_1_ economic development → **	**Y**_**1**_ **GDP-PER CAP-AAGR (%)**		**Alternative dependent variables**
**Y**_2_ **economic development**→**Inputs (X) and control (Z) factors** ↓	**Y**_2_ **Ln GDP per capita (2015 Constant)**		
Knowledge of finance (K) (0–7, high)	1.1	K	X_1_ financial literacy
Score for financial behavior (B): 0–9, high	1.2	B	
Financial attitudes (A) score (0–3, high)	1.3	A	
Financial literacy (FL)score (1-21, high)	1.4	FL	
Adults who actively manage their finances & create a budget (%)	1.5	Personal finance awareness	
Adults who compare prices on financial goods (%)	1.6		
Adults who understand simple and compound interest (%)	1.7		
Individuals in adulthood who comprehend the correlation between risk and return (RR) (%)	1.8	RR	
Adults who understand inflation (%)	1.9	INF	
Adults who understand RD (risk diversification) (%)	1.10	RD	
Outstanding loans with commercial banks	2.1A	A. Breadth of financial institutions	X_2_ SME credit financing
Commercial Bank - SME Loan Accounts	2.2A		
Non-Deposit MFI Loan Accounts	2.3A		
# of commercial bank branches per 1000 Km^2^	2.4B	B. Depth of financial institutions-branches	
# of MFI branches per 1000 Km^2^	2.5B		
# of commercial bank branches per 100,000 Adults	2.6B		
# of MFI branches per 100,000 Adults	2.7B		
# of loan accounts from all MFI per 1000 adults	2.8B		
# of branches of other deposit takers (ODT) per 100,000 Adults	2.9B		
SME deposit accounts (as a % of non-financial corporation (NFC) borrowers)	2.10C	C. SME access to credit	
SME loan accounts (as a % of NFC borrowers)	2.11C		
Outstanding SME Loan - Commercial Banks	2.12C		
SMEs with an outstanding loan or line of credit (%)	2.13C		
SMEs with a male owner with an account at a formal financial institution (%)	2.14C		
SMEs with an account at a formal financial institution (%)	2.15C		
SMEs with at least one female owner with an outstanding loan or line of credit (%)	2.16D	D. female SME share	
SMEs with at least one female owner with an account at a formal financial institution (%)	2.17D		
SMEs with outstanding credit who are required to provide collateral on loans (%)	2.18E	E. SME credit limitations	
SMEs with at least one female owner with outstanding credit who are required to provide collateral on loans (%)	2.19E		
SMEs with a male owner with outstanding credit who are required to provide collateral on loans (%)	2.20E		
# credit cards per 1000 adults	3.1	CC	X_3_ digital money–service and regulation
# debit cards per 1000 adults	3.2	DC	
# of ATMs per 1000 Km^2^	3.3	ATM	
# of ATMs per 100,000 Adults	3.4		
Registered mobile money agents (MMA) per 100,000 adults	3.5	MMA	
Value of Mobile & Internet Bank Transactions (during reference year) (%GDP)	3.6	Digital transactions	
Value of MMA Transactions (during reference year) (%GDP)	3.7		
Average # of MMA Transactions per active MMA	3.8		
Mobile Money Deployment (# of active services)	3.9		
Mobile Money regulatory index (0-100)	3.10	REG	
EC = Z_1_	Economic crisis events		Z_i_ macro factors and events
PE = Z_2_	Pandemic events		
CPI = Z_3_	Inflation		

Appendix 1 contains the details of data sources and the past research supporting the data elements.

## 5 Model results

As discussed in Section 2, several studies have underscored the importance of financial literacy and digital financial access in fostering SME growth and economic development. However, there is a need for a comprehensive analysis that employs robust statistical and machine learning techniques to quantify their impact accurately. This study utilizes Ordinary Least Squares (OLS), Random Forest (RFT), and Gaussian Mixture Model (GMM) methods to model the relationships between financial literacy, digital financial access, and economic outcomes. The study aims to identify the most effective approach for analyzing complex economic data by comparing these methods.

Principal Components (PCs) are computed by computing Eigen values and Eigen Vectors of covariance matrix. Let A be a square nXn matrix and X be a non-zero vector for which AX = λX for some scalar values λ. Then λ is known as the eigenvalue of matrix A and X is known as the eigenvector of matrix A for the corresponding eigenvalue. Sort the eigenvalues in descending order and sort the corresponding eigenvectors accordingly. Thus in **Tables 4**–**6** last two columns represent PC1 loading PC2 loading which are eigen vector of PC1 and PC2 arranged in decreasing order. The values in PC1 and PC2 columns show “explained variance” i.e., the term that gives us an idea of the amount of the total variance which has been retained by selecting the PCs instead of the original feature space. PCA employs a linear transformation that is based on preserving the most variance in the data using the least number of dimensions.

**Figure 4:**
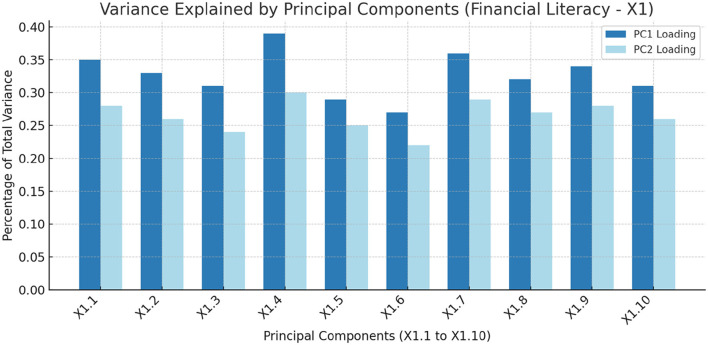
The Percentage of total variance explained by each principal component, highlighting the importance of each component in capturing the dataset's variability related to Financial Literacy (X_1_).

In Figure 4, Yellow bars indicates PC1 loading, and orange bars indicates PC2 loading in X_1_. X_1_ (Financial Literacy) principal components (PC) X_1.1_ to X_1.10_ are detailed in Table 3 Column 1 and 2 (a subset of Table 2).

**Table 3:** Feature importance of financial literacy (X_1_).

**Feature**		**Feature name**	**Principal component 1 loading**	**Principal component 2 loading**
Financial knowledge (K) score (0-7. high)	X_1.1_	K	0.35	0.28
Financial behavior (B) score (0-9. high)	X_1.2_	B	0.33	0.26
Financial attitudes (A) score (0-3. high)	X_1.3_	A	0.31	0.24
Financial literacy (FL) score (1-21. high)	X_1.4_	FL	0.39	0.30
Adults who actively budget/keep track of their money (%)	X_1.5_	Personal finance awareness (PFA)	0.29	0.25
Adults who shop around for financial products (%)	X_1.6_		0.27	0.22
Adults who understand simple and compound interest (%)	X_1.7_		0.36	0.29
Adults who understand the relationship between risk and return (RR) (%)	X_1.8_	RR	0.32	0.27
Adults who understand inflation (%)	X_1.9_	INF	0.34	0.28
Adults who understand RD (risk diversification) (%)	X_1.10_	RD	0.31	0.26

After conducting Principal Component Analysis (PCA) on variables X_1.1_ to X_1.10_, Figure 4 shows the percentage of variance explained by each component, highlighting their importance in explaining the variability related to financial literacy (X_1_).

The PCA on the financial literacy dataset (X_1.1_ to X_1.10_) provides key insights into the factors contributing to financial literacy among SMEs in the BRICS block. Principal Components 1 (PC1) and 2 (PC2) account for a significant portion of the data variability, with PC1 explaining the highest percentage (Yellow bars), indicating the dominant role of features (X_1.1_ to X_1.10_) in PC1.

The financial literacy score (X_1.4_) has the highest loading on PC1 (0.39), marking it as the most influential factor. This suggests that overall financial literacy, including various financial understanding aspects (X_1.4_), is crucial for SMEs. The loadings for “Adults who understand simple and compound interest” (X_1.7_) (0.36) and “Financial knowledge score” (X_1.1_) (0.35) also underscore the importance of basic financial concepts.

PC2 provides further insights, though with lower loadings. The financial literacy score (X_1.4_) remains significant, with a loading of 0.30, reaffirming its critical role in both PC1 and PC2. Table 3 details the loadings of each feature under the Financial Literacy category (X_1_), showing their relative importance in explaining the dataset's variance. This analysis emphasizes the various components of financial literacy, offering a comprehensive view of the most critical aspects for SMEs in the BRICS block. These insights are crucial for policymakers and educators aiming to improve financial literacy programs and support the economic growth of SMEs. By focusing on these key components, interventions and educational efforts can be prioritized, promoting a more financially competent SME sector capable of driving sustainable economic growth.

**Figure 5:**
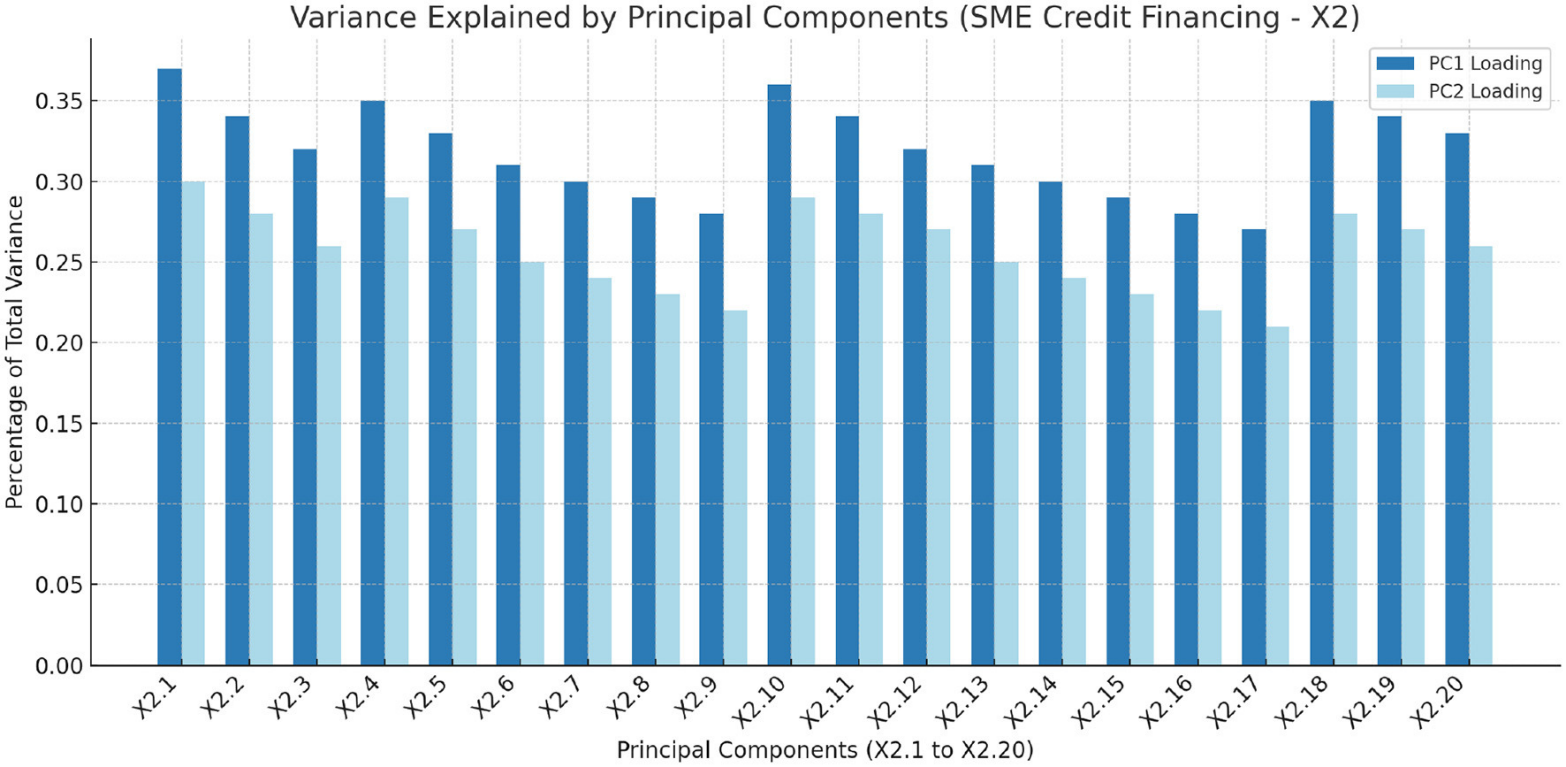
The Percentage of total variance explained by each principal component, highlighting the importance of each component in capturing the dataset's variability related to SME Credit Financing (X_2_) (these PCs are detailed in Table 4).

Figure 5 illustrates the percentage of total variance explained by each principal component, emphasizing the significance of each component in capturing the dataset's variability related to SME credit financing. This analysis provides insight into the underlying structure of the data, identifying the key dimensions that contribute to the overall variance.

Table 4 details the principal component loadings for each feature under the SME Credit Financing category (X_2_). The loadings for Principal Component 1 and Principal Component 2 highlight the contribution of each feature to the principal components, indicating their relative importance in explaining the variance within the dataset.

**Table 4:** Feature importance of (X_2_) SME credit financing.

**Feature**		**Feature name**	**Principal component 1 loading**	**Principal component 2 loading**
Outstanding loans with commercial banks	X_2.1_	A. Breadth of financial institutions	0.37	0.30
Commercial bank—SME loan accounts	X_2.2_		0.34	0.28
Non-deposit MFI loan accounts	X_2.3_		0.32	0.26
# of commercial bank branches per 1,000 Km^2^	X_2.4_	B. Depth of financial institutions-branches	0.35	0.29
# of MFI branches per 1,000 Km^2^	X_2.5_		0.33	0.27
# of commercial bank branches per 100.000 Adults	X_2.6_		0.31	0.25
# of MFI branches per 100.000 Adults	X_2.7_		0.30	0.24
# of loan accounts from all MFI per 1000 adults	X_2.8_		0.29	0.23
# of branches of other deposit takers (ODT) per 100.000 Adults	X_2.9_		0.28	0.22
SME deposit accounts (as a % of non-financial corporation (NFC) borrowers)	X_2.10_	C. SME access to credit	0.36	0.29
SME loan accounts (as a % of NFC borrowers)	X_2.11_		0.34	0.28
Outstanding SME loan–commercial banks	X_2.12_		0.32	0.27
SMEs with an outstanding loan or line of credit (%)	X_2.13_		0.31	0.25
SMEs with a male owner with an account at a formal financial institution (%)	X_2.14_		0.30	0.24
SMEs with an account at a formal financial institution (%)	X_2.15_		0.29	0.23
SMEs with at least one female owner with an outstanding loan or line of credit (%)	X_2.16_	D. Female SME share	0.28	0.22
SMEs with at least 1 female owner with an account at formal financial institution (%)	X_2.17_		0.27	0.21
SMEs with outstanding credit who are required to provide collateral on loans (%)	X_2.18_	E. SME credit limitations	0.35	0.28
SMEs with at least one female owner with outstanding credit who are required to provide collateral on loans (%)	X_2.19_		0.34	0.27
SMEs with a male owner with outstanding credit who are required to provide collateral on loans (%)	X_2.20_		0.33	0.26

Table 4 presents a principal component analysis (PCA) on the SME credit financing dataset, revealing key factors influencing SME credit access and financial health in the BRICS block. Principal Components 1 (PC1) and 2 (PC2) capture a significant portion of data variability, with PC1 explaining the most variance, highlighting the dominant features in PC1.

The highest loading on PC1 is for outstanding loans with commercial banks (X_2.1_) (0.37), indicating its pivotal role in SME credit access. Similarly, SME deposit accounts (X2.10) (0.36) and the number of commercial bank branches per 1000 Km2 (X_2.4_) (0.35) further emphasize the importance of financial institutions in SME credit financing.

PC2 adds further insights, with outstanding loans (X_2.1_) continuing to show significance (0.30), reaffirming its critical role in both components.

**Figure 6:**
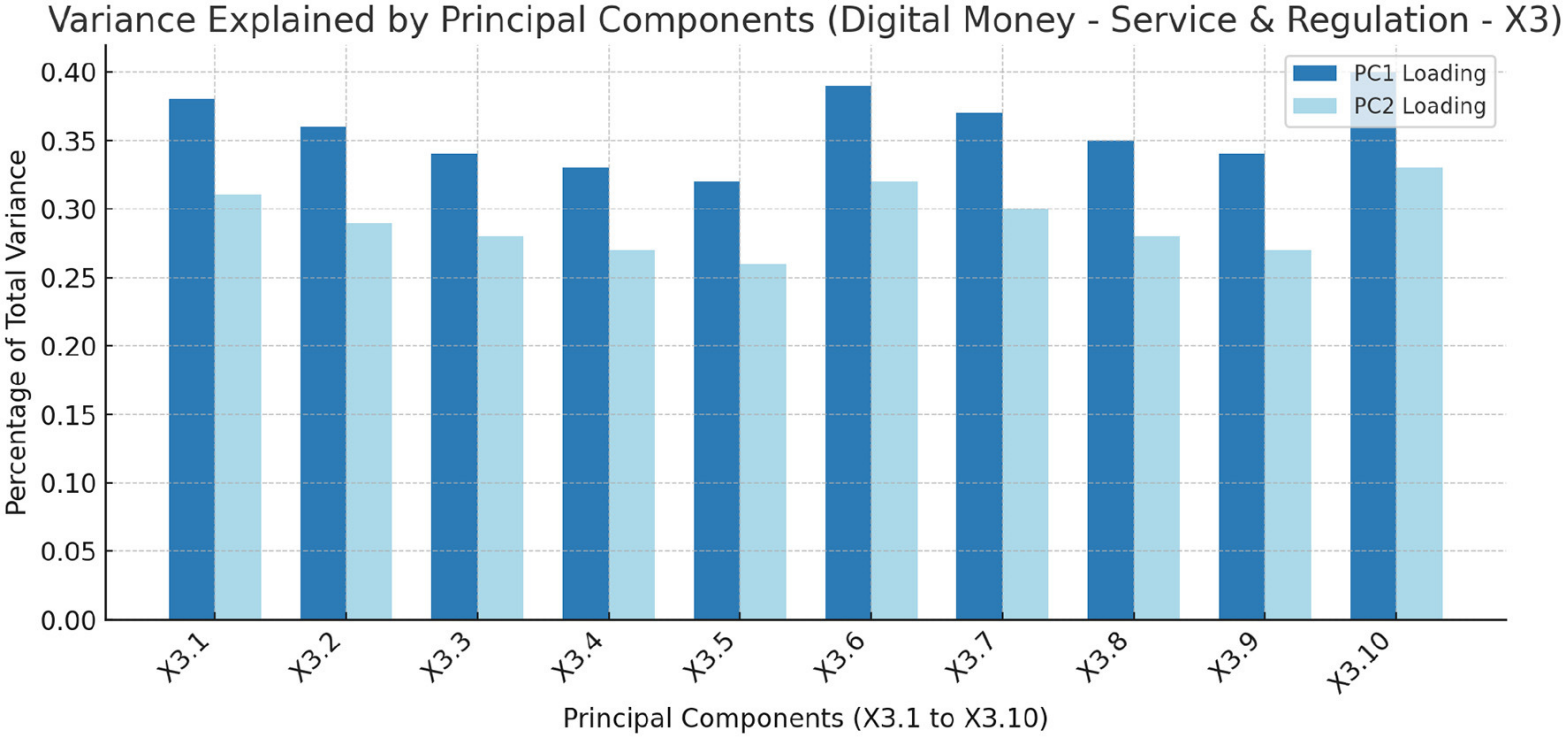
The Percentage of total variance explained by each principal component, highlighting the importance of each component in capturing the dataset's variability related to Digital Money–Service and Regulation (X_3_) (these values are detailed in Table 5).

Figure 6 illustrates the percentage of total variance explained by each principal component, emphasizing the significance of each component in capturing the dataset's variability related to digital money–service and regulation. This analysis provides insight into the underlying structure of the data, identifying the key dimensions that contribute to the overall variance. Table 5 details the principal component loadings for each feature under the Digital money–service and Regulation category (X_3_). The loadings for Principal Component 1 and Principal Component 2 highlight the contribution of each feature to the principal components, indicating their relative importance in explaining the variance within the dataset.

**Table 5:** Feature importance of (X_3_) digital money–service and regulation.

**Feature**		**Feature name**	**Principal component 1 loading**	**Principal component 2 loading**
# credit cards per 1,000 adults	X_3.1_	Credit card	0.38	0.31
# debit cards per 1,000 adults	X_3.2_	Debit card	0.36	0.29
# of ATMs per 1,000 Km^2^	X_3.3_	ATM	0.34	0.28
# of ATMs per 100,000 adults	X_3.4_		0.33	0.27
Registered mobile money agents (MMA) per 100,000 adults	X_3.5_	MMA	0.32	0.26
Value of mobile and internet bank transactions (during reference year) (%GDP)	X_3.6_	DIGITAL TRANSACTIONS	0.39	0.32
Value of MMA transactions (during reference year) (%GDP)	X_3.7_		0.37	0.30
Average # of MMA transactions per active MMA	X_3.8_		0.35	0.28
Mobile money deployment (# of active services)	X_3.9_		0.34	0.27
Mobile money regulatory index (0–100)	X_3.10_	REG	0.40	0.33

The principal component analysis (PCA) on the digital money-service and regulation dataset reveals key factors influencing the adoption and effectiveness of digital financial services in the BRICS block. Principal Components 1 (PC1) and 2 (PC2) capture significant data variability, with PC1 explaining the highest percentage, highlighting its dominant features.

The Mobile Money regulatory index (X_3.10_) has the highest loading on PC1 (0.40), underscoring the importance of regulatory frameworks in shaping digital financial services. Similarly, the Value of Mobile and Internet Bank Transactions (X_3.6_) (0.39) and the number of credit cards per 1000 adults (X_3.1_) (0.38) further emphasize the role of transaction volumes and credit access in digital service adoption.

PC2 offers additional insights, with the Mobile Money regulatory index (X_3.10_) remaining significant (0.33), reinforcing its critical role in both components.

Table 6 presents the coefficients and performance metrics for Ordinary Least Squares (OLS), Random Forest (RF), and Gaussian Mixture Model (GMM), analyzing the impact of various factors on Economic Development (Y1, Y2), with variables lagged by two periods to capture past influences.

**Table 6:** Coefficients of OLS, RF, and GMM for Y_1_ and Y_2_ (economic development).

**Independent variables (all lagged 2 periods)**	**Y**_**1**_ **[GDP-PER CAP-AAGR (%)]**			**Ln Y**_**1**_ **[Ln GDP per capita]**		
	**OLS**	**RFT**	**GMM**	**OLS**	**RFT**	**GMM**
α - Constant	0.19	0.19	0.25	0.64	0.19	0.15
β- Lagged 2 period dependent variable	0.12	0.24	0.14	0.08	−0.01	0.09
X_1_ Financial Literacy (FL)	0.15	0.21	0.17	0.31	0.29	0.05
X_2_ Access to Alternative Finance (AFF)	0.21	0.35	0.09	0.27	0.38	0.63
X_3_ Digitization	0.19	0.28	−0.03	0.53	0.57	0.24
GDP growth (annual %)	0.05	0.21	0.20	0.52	0.57	0.25
CPI - Inflation	0.15	0.24	0.21	0.32	0.28	0.12
Economic crises (EC) and pandemic events (PE)	0.09	0.23	0.10	0.36	0.28	0.62
**Diagnostics**						
R-squared	0.24	0.90	0.60	0.30	0.93	0.55
Adjusted R-squared	0.38	0.92	0.55	0.28	0.88	0.50
RMSE	1.00	0.12	0.50	0.85	0.10	0.45
AIC	400.34	250.20	350.45	390.20	200.15	345.50
BIC	420.50	270.10	360.35	410.30	220.40	355.25
Durbin-Watson statistic	1.20	2.50	1.60	1.25	2.90	1.55
VIF (Variance inflation factor)	5.00	1.50	3.50	4.85	1.40	3.20

In Table 6, model diagnostics in both Y_1_ and Y_2_ reveals that RFT specification outperforms OLS and GMM specifications, with higher R^2^, Adjusted R^2^, lowest root mean square error (RMSE), Akaike Information Criteria (AIC), Bayesian Information Criteria (BIC), DW Statistic around two, and VIF 2.8.

Further, model coefficients in RFT for Ln Y_1_ are consistently higher in magnitude (with exception of lagged dependent Ln Y_1_) than coefficients in RFT for Y_1_. This shows that the RFT specification is robust.

When comparing the model diagnostics between Y1 and Y2, all metrics are better for Y2. It has a higher R^2^ (0.93 vs. 0.9), lower RMSE (0.10 vs. 0.12), lower Akaike Information Criteria (AIC 200.15 vs. 250.2), Bayesian Information Criteria (BIC 220.40 vs. 270.1), and lower VIF (1.4 vs. 1.5). However, Y2′s adjusted R^2^ is slightly lower (0.88 vs. 0.92). This indicates that Y2′s specification is superior for modeling economic development, and RFT results will be used in the discussion.

These results demonstrate that the RFT method is the most reliable model for analyzing the impact of SME financial literacy, access to finance, and digitization on economic development in the expanded BRICS block. The superior performance of RFT highlights its ability to capture complex, non-linear relationships, making it valuable for policymakers and researchers seeking insights to foster SME growth and economic development in the region.

## 6 Results analysis and interpretation

The following are the key results from the RFT Model:

- **SME digitization (X**_**3**_**) had the highest positive impact on financial and economic development in the expanded BRICS bock**.

The feature values of digitization are of the highest magnitude (0.57) signifying the importance of digitalization of SMEs. Higher the digitization, the economic development grows by 0.57 times in the expanded BRICS block. This validates our third hypothesis that digitization fosters economic development. This finding is consistent with Khera et al. (2021) who conclude that the interplay among financial literacy, credit accessibility, and digital transformation is a crucial element in the sustainable expansion and competitiveness of SMEs in the digital era. Their narrative is that Digital financial literacy does not stand in isolation but is part of a broader ecosystem that includes digital infrastructure, regulatory frameworks, and the availability of digital financial services. The integration of these elements can catalyze the transformation of SMEs from traditional operations to more innovative and scalable business models. As such, SMEs' engagement with digital financial services is expected to enhance their financial inclusivity, thereby reducing the traditional barriers to credit access and financial services (Khera et al., 2021).

- **SME access to alternative finance (X**_**2**_**) is also positively related with Financial and Economic Development in expanded BRICS block (Ln Y**_**1**_**)**.

Consistent with knowledge-based and resource-based theories, SMEs' access to alternative finance has a feature weight of 0.38. This implies that with higher SMEs' access to alternative finance, economic development grows by 0.38 times in the expanded BRICS block. This validates our second hypothesis that SME's access to credit financing positively impacts economic development. These findings are consistent with (Dewi and Yurniwati, 2018) who concluded that funding access is a critical component of small businesses' long-term success. Because it enables them to experiment with new ideas, launch creative projects, and become more prepared to take advantage of opportunities. It is also consistent with Nzibonera and Waggumbulizi (2020) who conclude that financial access promotes profitability and growth while ensuring stability and sustainability. Small companies that take out external debt are better positioned for growth, and these companies' ability to obtain alternative funding is crucial to their success (Patrick et al., 2021).

- **SME financial literacy (X**_**1**_**) is positively associated with Financial and Economic Development in the expanded BRICS block (Ln Y**_**1**_**)**.

The improvement in financial literacy of SMEs results in 0.27 times increased economic development in the expanded BRICS block. This validates our first hypothesis that SME's financial literacy positively ensures sustainable economic development. This result is consistent with the small business literature, where there is documented proof of how owner-managers' knowledge of finance influences the companies' profitability (Eniola and Entebang, 2016; Kimunduu et al., 2016; Kizza, 2019). The influence of financial literacy on loan availability for entrepreneurs is related to this effect (Dewi and Yurniwati, 2018); additionally, the viability of small businesses owned and controlled by entrepreneurs is impacted by formal credit availability (Kalaieesan, 2021; Patrick et al., 2021).

- **Control factors like sustained GDP growth (0.57) also ensures sustained Economic Development of expanded BRICs block followed by higher inflation (0.28) and pandemic events (0.28)**.

The implication of these control factors is that intervention by the respective government to reduce the level of disruption and hardship of SMEs during inflationary and pandemic events substantially helps in the sustained economic development of the expanded BRICS block. These findings are consistent with Kraus et al., 2022; Marinko et al., 2021; Xie et al., 2022, who report that SMEs are being forced to modify their business models and strategies due to various factors, comprising probable recession, related uncertainty, and inflation. These findings are also consistent with the digitization theory, that changes in the global market and socioeconomic landscape are forcing SMEs to go digital. The current global issues serve as an example of how numerous business risks and uncertainties, such as pandemics, can disrupt global supply chains and accelerate the speed of digital transformation (Thanos et al., 2021).

### 6.1 Predicted values

The foregoing results highlight RFT's robustness in capturing the complex, non-linear relationships within the data, making it the most reliable and accurate method for analyzing the impact of financial literacy, access to finance, and digitization on economic growth and development in the expanded BRICS block.

Table 7 compares the predicted and actual means for Y1 and Y2 for 2022 and 2023, along with their respective RMSE values, which indicate prediction accuracy. Lower RMSE values suggest higher model accuracy.

**Table 7:** Predicted and actual means of expanded BRICS economic development.

**Variable**	**Predicted mean**	**Actual mean**	**RMSE**
Y_1_ (GDP-PER CAP-AAGR (%))	4.3	4.8	0.55
Y_2_ (Ln GDP per capita)	9.2	8.7	0.45

For Y1, the model predicts an average annual growth rate of 4.3% for the expanded BRICS countries, closely matching the actual mean of 4.8%, with an RMSE of 0.55, demonstrating good accuracy in predicting economic development.

Similarly, for Y2, the predicted average is 9.2, compared to the actual mean of 8.7, with an RMSE of 0.45, signifying accurate predictions. In terms of constant GDP per capita (2015 values), the model predicts an average of $9987 for 2023-24, while the actual value is $6003.

The low RMSE values for both Y1 and Y2 show the model's strong predictive performance, with the close alignment of predicted and actual means confirming the reliability of the RFT model for economic planning and policy decisions.

## 7 Conclusion, implications, limitations and directions for future research

### 7.1 Conclusion

With the focus on the UN's SDG 2030 Target by 2030 and financial inclusivity through alternative finance for SMEs, this research investigated whether and to what degree SME's financial literacy, SME's access to alternative credit financing and SMEs' digitization are linked to expanded BRICS' economic development. The research fills the gap of missing information about how digitization is impacting SME's financial literacy and access to credit finance and in turn, fostering economic development in the expanded BRICS Three plausible hypotheses were framed to support the research objectives. Principal Component Analysis (PCA) helped to simplify complex datasets and reduce dimensionality and collinearity issues in the data set over 2000–2023. Each of the three methodologies—OLS, GMM, and Random Forest Tree (RFT) brings distinct advantages to the analysis of SMES digitalized financial awareness and access to alternative credit finance during uncertain economic environments, including recession and pandemic events. digitally enabled microfinance. While OLS offers simplicity and a good starting point for linear relationships, GMM addresses endogeneity in dynamic settings. RFT complemented these by capturing complex, non-linear interactions, and variable (feature) importance, offering a comprehensive toolkit for understanding the multifaceted impacts of digital financial services on income equality.

When viewed from the perspective of model diagnostics, the ML model RFT proved to have a superior model specification to traditional OLS and GMM models. The RFT model findings indicated that the feature values of digitization are of the highest magnitude (0.57) signifying the importance of digitalization of SMEs to ensure sustainable economic development. The higher the digitization in the expanded BRICS block, the more economic development will grow by 0.57 times in the expanded BRICS block.

Digitization is followed by SMEs' access to alternative finance with a weight of 0.38. This implies that the higher the SMEs' access to alternative finance, the more economic development grows by 0.38 times in the expanded BRICS block.

Equally important is the financial literacy of SMEs as depicted through various sub-indices. The improvement in SMEs' financial literacy results in 0.27 times increased economic development in the expanded BRICS block.

Control factors like sustained GDP growth (0.57) also ensure sustained Economic Development of expanded BRICs block, followed by higher inflation (0.28) and pandemic events (0.28) which results in intervention by the respective government to reduce the level of disruption and hardship of SMEs during inflationary and pandemic events.

### 7.2 Policy implications

1. Considering the RFT results, *policymakers and stakeholders should consider leveraging advanced analytical techniques like RFT to better understand and address the needs of SMEs*. enhancing financial literacy and expanding digital financial access can significantly contribute to the sustainable economic growth of the BRICS nations, fostering a more inclusive and resilient economic environment.

2. Policymakers should *prioritize the development and implementation of educational programs aimed at improving digital financial literacy among SMEs*. this can help bridge the knowledge gap, enabling SMEs to better utilize digital financial services and tools, thereby enhancing their ability to access alternative finance and contribute to economic development.

The government and regulatory bodies in the BRICS countries have undertaken various initiatives to enhance the adoption of FinTech and develop a resilient, responsible, and facilitating digital financial infrastructure and ecosystem, to create an enabling regulatory framework to promote digital and financial literacy and support consumer and data protection. On account of all these efforts, digitalization has progressed significantly in all the BRICS countries.^4^ A few real-world examples of financial literacy and digital inclusion in BRICS nations are as below to justify the policy implications for policymakers.

#### 7.2.1 Financial literacy

As illustrated in the chart in the introduction section, digital financial literacy combines the skills needed to navigate financial services with the skills to use digital technologies.

#### 7.2.2 Digital literacy and inclusion

Digital wallets (also called e-wallets) offer a range of financial services, including money transfers and payments. Many financial institutions also have digital savings accounts, which enables a user to manage their finances online. Recognizing the importance of digital ID, the BRICS countries have taken significant steps in this direction. Some of the success stories in BRICS are provided below as examples.

- Brazil launched an app in 2020 that combines the social security card *(Cadastro de Pessoas F*í*sicas)* and driving license as part of its digital identification and citizen service delivery plan. The digital versions of the documents available through the new app are validated through a QR Code.
- Russia launched the Digital Profile project in 2020, as a part of the Unified System of Identification and Authentication (USIA) which allows citizens to manage their digital consent for providing their personal data to companies in real-time, simplifying access to data, making their provision faster and more transparent, as well as improving the quality and lowering the costs of services. It is used for issuing loans and signing car insurance agreements (CMTPL and Casco^5^), and was expanded to cover microfinance institutions and financial platform/marketplace operators. Along with the Unified biometric system it provides for the secure identification and authentication of citizens and exchange of reliable customers' data from government sources.
- India's Aadhaar platform is the world's largest digital identity platform with more than one billion enrolments^6^ It is linked to services such as UPI, e-Sign, and e-KYC; and is used to verify identity anywhere, helping in seamlessly accessing payments and Government services. Additionally, “Digital KYC” and “Equivalent e-Documents,” were included through the amendment of Prevention of Money-laundering Rules^7^ and RBI's Know Your Customer (KYC) Directions.
- As part of China's digital ID system, a peer-to-peer facial recognition app called Zhen Ni (The Real You) was launched in 2019. The country's digital ID was also integrated with the online platform WeChat, in a bid to allow users to synchronize their national ID cards with the app and use their phones as IDs to buy train tickets or book hotels.
- South Africa plans to integrate its biometric database to boost digital ID, and the government has put out the new draft Official Identity Management Policy for public comments in December 2020.^8^
- Austria has implemented the “Digital Austria” initiative, which focuses on digital transformation across various sectors, including SMEs. The initiative promotes the use of e-government services and digital identities to streamline business operations and improve access to government services. The Austrian government has also introduced the “KMU DIGITAL” program, providing financial support and guidance to SMEs for their digitalization efforts. This program has successfully helped many small businesses integrate digital technologies, enhancing their competitiveness and access to new markets. The BRICS countries could learn from Austria's approach by creating similar support systems that incentivize SMEs to adopt digital tools, thereby fostering innovation and economic growth across the block.

The BRICS authorities have to ensure the security, data protection and transparency of the system and link to various payment platforms with negligible rejection rates.

### 7.3 Practical implications

The study persuades owner-managers to recognize the value of SME financial literacy in accessing formal credit and boosting sustainability. The study finding also encourages SME managers to work closely with higher educational institutions since they are the key to gaining financial literacy through training. For sustainability and a competitive economy, financial literacy is not a luxury for owner-managers of SMEs since it reduces information asymmetry when they seek formal credit from financial institutions. Therefore, SMEs should prioritize improving their financial literacy to make informed financial decisions and increase their chances of accessing formal credit financing.

The BRICS block can create a more robust SME ecosystem that can better survive global crises and help the most vulnerable areas of their economies by combining their resources and expertise.

In the past few years, the BRICS countries have made considerable progress in leveraging digital technologies to achieve higher levels of financial inclusion. But the journey does not end with mere inclusion itself; financial literacy and education-based empowerment of the customer remain the most vital cog in the system. A well-informed and empowered customer is the most crucial component of a sustainable and stable financial ecosystem. The BRICS countries are proactively pursuing this agenda, given the high levels of financially and digitally vulnerable sections of the population. The ‘Strategy for BRICS Economic Partnership 2025′ acknowledges that member countries are not at the same levels of digital development and there is a need to focus on addressing the digital divide and ensuring shared benefits of digitalization. The BRICS countries have a high potential for cooperation in bridging the digital divide and promoting financial literacy. Given the associated challenges, it is necessary to continuously evaluate the impact of the actions undertaken and accordingly adapt and reform our strategies.

### 7.4 Societal implications

The importance of international collaboration among BRICS blocks in sharing best practices and resources is vital for enhancing the efficacy of digitization, especially during challenging times like pandemics. Such collaboration offers a platform for these nations to exchange innovative strategies and solutions that have proven effective in their respective contexts. This includes sharing technological advancements in digital banking, risk assessment models tailored for pandemic conditions, and policies that support financial inclusivity. While efforts are being made to deepen and expand the reach of digital financial services, BRICS countries have to collaboratively work toward a faster, more convenient, affordable, and secure payment system, both for the individual consumer and the small merchants and businesses. The key to addressing the digital divide, within and across countries, is to expand access to technology (mobile phones, the internet, electricity, and digital ID), enable online-offline integration, design appropriate financial services and financial and digital literacy. The BRICS members are trying innovative approaches including inter alia open banking, differentiated banks, and guidelines to facilitate engagement of the low-income households, small businesses, and the informal sector. The BRICS countries have a significant scope of learning from each other, and the report provides a toolkit for connecting the global guidelines evolving on the current issues with the on-ground efforts being taken by the BRICS. Thus, the countries can align their strategies with global commitments while learning from their peers.

Sustained policy interventions to promote the SME sector through various infrastructural investments are critical to promote entrepreneurial startups to improve the earning potential of women and the needy to improve their earning potential to reduce income inequality.

The increased usage of digital payment systems facilitated financial transactions during the COVID-19 pandemic and the subsequent lockdowns, social distancing and no-contact protocols. There was a 42 percent increase in global cashless payments (PwC study, 2021). Furthermore, there was a cross-generational shift to digital channels with older people (56 years and above) increasingly accessing digital payment modes during this period. The private sector, especially small businesses, increased their presence online and gave an impetus to the digitalization of payments. The private players are also spearheading innovation in the retail digital payment space and fostering financial inclusion. These increased retail transactions along with the digital direct transfer of COVID-19-related social benefits from government to individuals and SMEs, expanded the ambit of financial inclusion.

While the COVID-19 pandemic has brought about a change in thinking in the way banking and payments are being used, the authorities must address the challenges of the digital divide, amplified by a lack of digital access and digital literacy. There was enhanced recourse to using social media and mass media (including local TV channels and radio), for dissemination of financial education. Greater financial literacy and education, together with sound consumer protection mechanisms, will ensure that people at the bottom of the pyramid are empowered to take informed financial decisions.

### 7.5 Limitations

Some limitations and concerns with the data at the national level must be resolved in future revisions before it can be considered complete. For instance, for the newly added seven economies to the existing BRICS block, independent and macro data from 2000-2015 were patchy, requiring careful data interpolation wherever they were found missing. Improved availability of data from these new economies is particularly important to identify potential synergies available in these and the BRICS block that can be shared by all economies wherever needed for improved income equality of the vulnerable populace.

### 7.6 Directions for future research

While the PCA is a practical methodology for reducing dimensionality and collinearity issues, IMF has developed a new Financial Development Index (FDI) to measure financial development (FD). Future studies can use these indices as alternative output indicators to check the robustness of current model results. Secondly, the research work is at the BRICS block level. It would be useful to perform individual countrywide granular evaluations of the research objectives to provide granularity to the specific policy interventions by the respective governments.

This study contributes to the growing body of literature on SME development and economic modeling by demonstrating the efficacy of machine learning methods in financial research. Future research could further explore integrating other advanced techniques and their application to different economic contexts, providing deeper insights into the dynamics of SME growth and development.

## Data Availability

The original contributions presented in the study are included in the article/supplementary material, further inquiries can be directed to the corresponding authors.

## Footnotes

^1^Lauer, K. and Lyman, T., (2015) “Digital Financial Inclusion: Implications for Customers, Regulators, Supervisors, and Standard-Setting Bodies”, Consultative Group to Assist the Poor (CGAP), February.

^2^The average FD for all nations is 0.23; the average FD for advanced markets (AM) is 0.57; the average FD for emerging markets (EM) is 0.23; and the average FD for low-income and developing countries (LIDC) is 0.11 (Svirydzenka, 2016).

^3^ https://data.imf.org/?sk=f8032e80-b36c-43b1-ac26-493c5b1cd33b

^4^BRICS Digital Financial Inclusion Report, August 2021. This report is developed by the RBI with collaboration and inputs from other BRICS central banks, i.e., Central Bank of Brazil (BCB), Central Bank of the Russian Federation (Bank of Russia, BoR), People's Bank of China (PBoC) and South African Reserve Bank (SARB).

^5^CMTPL - Compulsory Motor Third Party Liability and Casco - Comprehensive and Collision car insurance.

^6^ https://www.digitalindia.gov.in/ebook/deity/page4.php

^7^Prevention of Money-laundering (Maintenance of Records) Amendment Rules, 2015.

^8^ https://www.gov.za/sites/default/files/gcis_document/202101/44048gon1425.pdf
