# Supplementary material for: How critical is SME financial literacy and digital financial access for financial and economic development in the expanded BRICS block?
Source: Front Big Data. 2024 Dec 19;7:1448571. doi: 10.3389/fdata.2024.1448571 (PMC11693673; doi:10.3389/fdata.2024.1448571)
Supplement: Supplementary file 2 [file Data_Sheet_1.docx]

**Appendix-1 Measurement and sources details of variables**

| **Variable** | **Data Sources** | **Previous research** |
| --- | --- | --- |
| Alternative Economic Development & Financial Development Variables. (Dependent Variable)  **Y_1_ = GDP-PER CAP-AAGR (%)**  **LnY_1_ = Natural log -Ln GDP per capita (2015 Constant)** | World Development Indicators | Agyei, 2018.  Bernanke, Gertler, & Gilchrist 1999. |
| **Y_2_ = FINANCIAL DEVELOPMENT INDEX-FDI** | Financial Development Index  <https://data.imf.org/?sk=f8032e80-b36c-43b1-ac26-493c5b1cd33b> | Sahay et al., 2015 |

**Independent Variables (X_ijt_)**

1. **X_1_ FINANCIAL LITERACY**

| **Sub-indicators** | **Data Sources** | **Previous research** |
| --- | --- | --- |
| 1. Financial knowledge (K) score (0-7, high) | OECD International Survey of Adult Financial Literacy: <https://www.oecd.org/financial/education/oecd-infe-2020-international-survey-of-adult-financial-literacy.pdf> | OECD, 2020.  Agyapong & Attram, 2019.  Zhao, 2019.  Widiyati et al., 2018.  Ali et al., 2018.  Eniola & Entebang, 2016.  Putra et al., 2021.  Owusu et al., 2021. |
| 1. Financial behavior (B) score (0-9, high) |  |  |
| 1. Financial attitudes (A) score (0-3, high) |  |  |
| 1. Financial literacy (FL)score (1-21, high) |  |  |
| 1. Adults who actively budget/ keep track of their money (%) |  |  |
| 1. Adults who shop around for financial products (%) |  |  |
| 1. Adults who understand simple and compound interest (%) |  |  |
| 1. Adults who understand the relationship between risk and return (RR) (%) |  |  |
| 1. Adults who understand inflation (%) |  |  |
| 1. Adults who understand RD (risk diversification) (%) |  |  |

II. **X_2_ SME CREDIT FINANCING**

| **Sub-indicators** | **Data Sources** | **Previous research** |
| --- | --- | --- |
| 1. Outstanding loans with commercial banks | IMF Financial Access Survey Data | Dewi et al., 2018.  Eniola & Entebang, 2016.  Kimunduu et al., 2016.  Kizza, 2019.  Kalaieesan, 2021.  Patrick et al., 2021.  Ye & Kulathunga, 2019a.  Nzibonera & Waggumbulizi, 2020.  Buchdadi et al., 2020  Khan et al., 2022.  Babajide et al., 2021.  Delic et al. (2016). |
| 1. Commercial Bank - SME Loan Accounts |  |  |
| 1. Non-Deposit MFI Loan Accounts |  |  |
| 1. # of commercial bank branches per 1000 Km^2^ |  |  |
| 1. # of MFI branches per 1000 Km^2^ |  |  |
| 1. # of commercial bank branches per 100,000 Adults |  |  |
| 1. # of MFI branches per 100,000 Adults |  |  |
| 1. # of loan accounts from all MFI per 1000 adults |  |  |
| 1. # of branches of other deposit takers (ODT) per 100,000 Adults |  |  |
| 1. SME deposit accounts (as a % of non-financial corporation (NFC) borrowers) |  |  |
| 1. SME loan accounts (as a % of NFC borrowers) |  |  |
| 1. Outstanding SME Loan - Commercial Banks |  |  |
| 1. SMEs with an outstanding loan or line of credit (%) |  |  |
| 1. SMEs with a male owner with an account at a formal financial institution (%) |  |  |
| 1. SMEs with an account at a formal financial institution (%) |  |  |
| 1. SMEs with at least one female owner with an outstanding loan or line of credit (%) |  |  |
| 1. SMEs with at least one female owner with an account at a formal financial institution (%) |  |  |
| 1. SMEs with outstanding credit who are required to provide collateral on loans (%) | IMF Financial Access Survey Data | Fourati & Affes, 2013.  Karyadi & Rizki, 2018. |
| 1. SMEs with at least one female owner with outstanding credit who are required to provide collateral on loans (%) |  |  |
| 1. SMEs with a male owner with outstanding credit who are required to provide collateral on loans (%) |  |  |

1. **X_3_ Digital Money - Service & Regulation**

In 2018, Global Survey of Mobile Access (GSMA) revised definition of mobile money. Now, a service is considered a mobile money service if it meets the following criteria:

- A mobile money service includes transferring money and making and receiving payments using the mobile phone.
- The service must be available to the unbanked, e.g. people who do not have access to a formal account at a financial institution.
- The service must offer a network of physical transactional points which can include agents, outside of bank branches and ATMs, that make the service widely accessible to everyone.
- Mobile banking or payment services (such as Apple Pay and Google Wallet) that offer the mobile phone as just another channel to access a traditional banking product are not included.
- Payment services linked to a traditional banking product or credit card, such as Apple Pay and Google Wallet, are not included."

| **Sub-indicators** | **Data Sources** | **Previous research** |
| --- | --- | --- |
| 1. # credit cards per 1000 adults | WDI &  IMF Financial Access Survey (FAS) | Kraus et al., 2022.  Škare and Soriano, 2021.  Xie et al., 2022.  Matarazzo et al., 2021.  Müller et al., 2020.  Khin & Ho, 2018. Parise et al., 2016.  Gal et al., 2019. Hai, et al., 2020.  Atina Shofawati, 2019.  Garzoni et al., 2020.  Kraus et al., 2022 |
| 1. # debit cards per 1000 adults |  |  |
| 1. # of ATMs per 1000 Km^2^ |  |  |
| 1. # of ATMs per 100,000 Adults |  |  |
| 1. Registered mobile money agents (service provider) (MMA) per 100,000 adults |  |  |
| 1. Value of Mobile & Internet Bank Transactions (during reference year) (%GDP) |  |  |
| 1. Value of MMA Transactions (during reference year) (%GDP) |  |  |
| 1. Average # of MMA Transactions per active MMA |  |  |
| 1. Mobile Money Deployment (# of active services) |  |  |
| 1. Mobile Money regulatory index (0-100) |  |  |

1. **Z_i_ Control variables**

| **Variable** | **Data Sources** | **Previous research** |
| --- | --- | --- |
| 1. GDP growth (annual %) | World Development Indicators (WDI)  World Bank national accounts data, | Zhang & Naceur, 2019. |
| 1. CPI - Inflation | WDI database |  |
| 1. Economic Crises (EC) and Pandemic Events (PE) | WHO database | Papadopoulos, Baltas, & Balta, 2021; |
